# Supplementary figures and images for: Analysis of low-density lipoprotein receptor gene mutations in a family with familial hypercholesterolemia
Source: PLoS One. 2024 Oct 11;19(10):e0310547. doi: 10.1371/journal.pone.0310547 (PMC11469539; doi:10.1371/journal.pone.0310547)

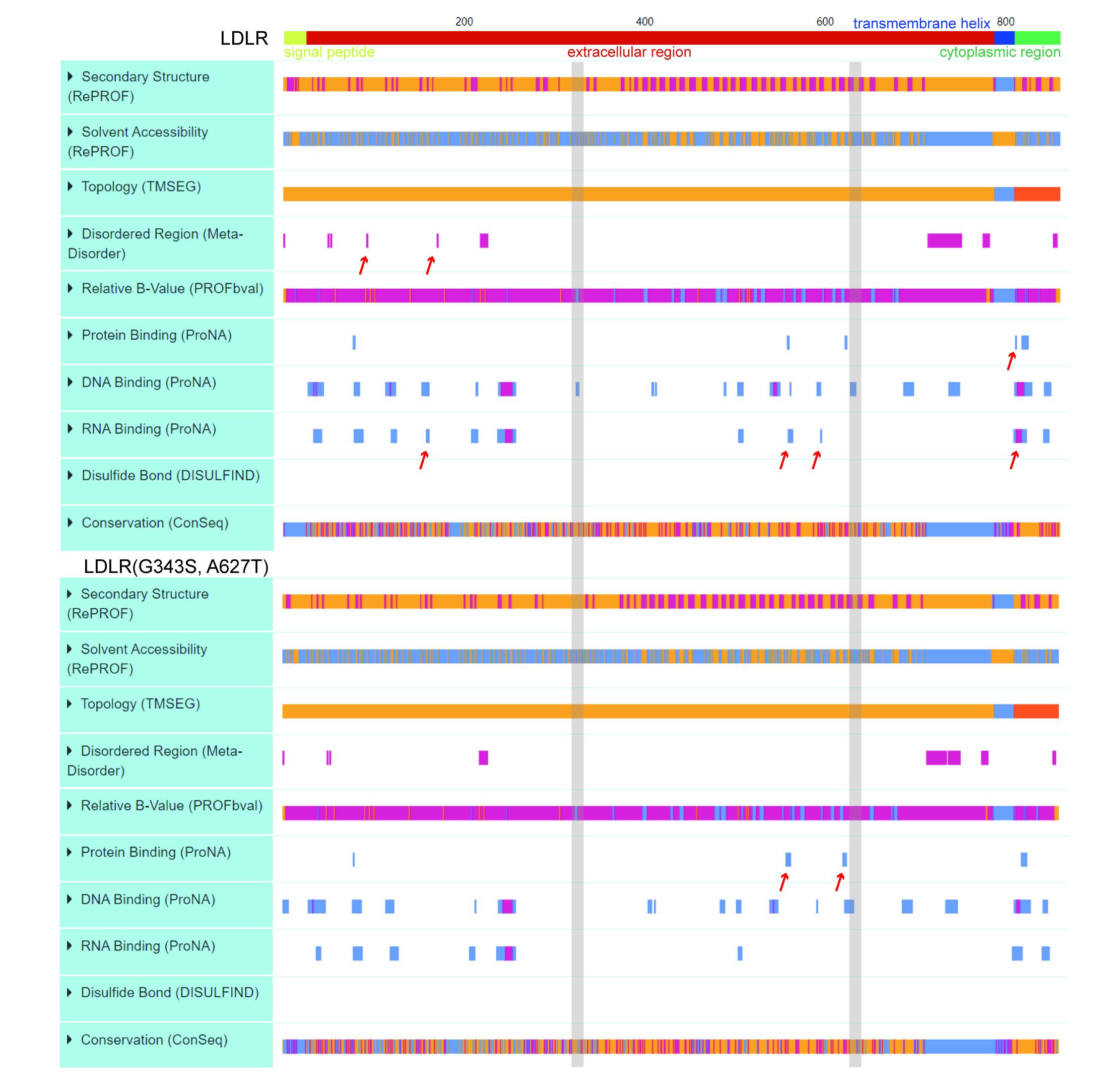

Supplement: S1 Fig — Functional domain prediction for wild-type (top) and mutant (bottom) using Predictprotein (https://predictprotein.org/). The shaded area represents the location of the mutation; the red arrows indicate regions where wild-type and mutants vary significantly in functional domains. (TIF) [file pone.0310547.s001.tif]
