# Supplementary material for: Analysis of low-density lipoprotein receptor gene mutations in a family with familial hypercholesterolemia
Source: PLoS One. 2024 Oct 11;19(10):e0310547. doi: 10.1371/journal.pone.0310547 (PMC11469539; doi:10.1371/journal.pone.0310547)
Supplement: Attachment — Submitted filename: Response to Reviewers.docx [file pone.0310547.s002.docx]

**Dear editors,**

Thank you for your letter and for the reviewers’ comments concerning our manuscript entitled “Analysis of low-density lipoprotein receptor gene mutations in a family with familial hypercholesterolemia”(Submission ID number PONE-D-24-12858). Those comments are all valuable and very helpful for revising and improving our manuscript, as well as the important guiding significance to our researches. We have studied comments carefully and have made correction which we hope meet with approval. Revised portion are marked in red in the manuscript. The main corrections in the manuscript and the responds to the reviewer’s comments are listed below this letter.

We would like to express our great appreciation to you and reviewers for comments on our manuscript. If you have any questions, please do not hesitate to contact me.

With best wishes,

Yours sincerely,

Fan Lin

Jie-Wei Luo

Hong-Li

2024-7-10

Journal Requirements:

When submitting your revision, we need you to address these additional requirements.

1. Please ensure that your manuscript meets PLOS ONE's style requirements, including those for file naming. The PLOS ONE style templates can be found at

https://journals.plos.org/plosone/s/file?id=wjVg/PLOSOne_formatting_sample_main_body.pdf and

<https://journals.plos.org/plosone/s/file?id=ba62/PLOSOne_formatting_sample_title_authors_affiliations.pdf>

**Re: Thank you very much for your comments, we have already revised the manuscript and file name according to PLOS ONE's style requirements.**

2. Thank you for submitting the above manuscript to PLOS ONE. During our internal evaluation of the manuscript, we found significant text overlap between your submission and previous work in the [introduction, conclusion, etc.].

We would like to make you aware that copying extracts from previous publications, especially outside the methods section, word-for-word is unacceptable. In addition, the reproduction of text from published reports has implications for the copyright that may apply to the publications. Please revise the manuscript to rephrase the duplicated text, cite your sources, and provide details as to how the current manuscript advances on previous work. Please note that further consideration is dependent on the submission of a manuscript that addresses these concerns about the overlap in text with published work. We will carefully review your manuscript upon resubmission and further consideration of the manuscript is dependent on the text overlap being addressed in full. Please ensure that your revision is thorough as failure to address the concerns to our satisfaction may result in your submission not being considered further.

**Re: Thank you very much for your comments, we have completely revised the** **introduction, discussion and conclusion sections in the manuscript.**

3. We note that the grant information you provided in the ‘Funding Information’ and ‘Financial Disclosure’ sections do not match.

When you resubmit, please ensure that you provide the correct grant numbers for the awards you received for your study in the ‘Funding Information’ section.

**Re:** **Sorry, we have corrected the grant numbers and ensured that all information now accurately reflects the funding sources for our study.**

4. PLOS ONE now requires that authors provide the original uncropped and unadjusted images underlying all blot or gel results reported in a submission’s figures or Supporting Information files. This policy and the journal’s other requirements for blot/gel reporting and figure preparation are described in detail at https://journals.plos.org/plosone/s/figures#loc-blot-and-gel-reporting-requirements and https://journals.plos.org/plosone/s/figures#loc-preparing-figures-from-image-files. When you submit your revised manuscript, please ensure that your figures adhere fully to these guidelines and provide the original underlying images for all blot or gel data reported in your submission. See the following link for instructions on providing the original image data: https://journals.plos.org/plosone/s/figures#loc-original-images-for-blots-and-gels.

In your cover letter, please note whether your blot/gel image data are in Supporting Information or posted at a public data repository, provide the repository URL if relevant, and provide specific details as to which raw blot/gel images, if any, are not available. Email us at plosone@plos.org if you have any questions.

**Re:** **Thank you for your guidance regarding the submission requirements for blot and gel results. We have reviewed PLOS ONE's updated policies on reporting requirements for blots and gels and have prepared our manuscript accordingly.**

5. Your ethics statement should only appear in the Methods section of your manuscript. If your ethics statement is written in any section besides the Methods, please move it to the Methods section and delete it from any other section. Please ensure that your ethics statement is included in your manuscript, as the ethics statement entered into the online submission form will not be published alongside your manuscript.

**Re:** **Thank you for your comments, we have deleted ethics statement in other section besides the Methods.**

6. Please include complete captions for your Supporting Information files at the end of your manuscript, and update any in-text citations to match accordingly. Please see our Supporting Information guidelines for more information: <http://journals.plos.org/plosone/s/supporting-information>.

**Re: Thank you for your comments,** **since we have revised the manuscript, there is currently no supporting information in the manuscript.**

Review Comments to the Author

Reviewer 1

The authors reported variants in LDLR gene in a Chinese family with FH. In silico analysis and functional assays were perform. Some major issues need to be addressed.

**Major comments:**

1.Row 27 (and in whole body of manuscript): LDLR - gene (genes) name should be writen in italics.

**Re:** **Thank you for your comments, we have already revised it in whole body of manuscript.**

2.Row 32: Rather genotype-phenotype associations“ than „genetic-phenotypic associations“.

**Re:** **Thank you for your comments, we have already revised it.**

3.Rows 49-51: Conclusions“: Presented information are well known for many years and cannot be presented as conclusions of this study. Please, completely revise the text.

**Re:** **Thank you for your comments, we have already revised the conclusions section completely.**

4.Row 69: Rather “caused by pathogenic variations” than “caused by variations”.

**Re:** **Thank you for your comments, we have already revised it.**

5.Row 77: Rather “common population” than “average people”.

**Re:** **Thank you for your comments, we have already revised it.**

6.Row 86: The author is writing about the apolipoprotein APOB, not the gene, so italics cannot be used.

**Re:** **Thank you for your comments, we have already revised it.**

7.Row 93: 1951? Joseph Leonard Goldstein (* 18. April 1940) and Michael Stuart Brown (* 13. April 1941), so at the age of 11 and 10? – reference [17] is from 1974.

**Re: Very sorry, the correct statement is 1974, rather than 1951. We apologize for our mistake and have already revised it.**

8.Row 176: Use italics for LDLR.

**Re:** **Thank you for your comments, we have already revised it.**

9.Row 176: Which “Target genes…”.

**Re:** **Thank you for your comments, the target gene refers to the LDLR gene carrying the c.1027G>A mutation and c.1879G>A mutation.**

10.Row 218: …of PROS1? Why PROS1? This is the reason: PMID: 34496879? In my opinion, you overwrote (copy/pasted) your older text.

**Re: Very sorry, we accidentally pasted our older text (PMID: 34496879). We apologize for our carelessness and we have already revised it.**

11.Row 264: “Simon criteria” are “Simon Broome criteria”, “Dutch Network Diagnostic Criteria” are “Dutch Lipid Clinic Network criteria”.

**Re:** **Thank you for your comments, we have already revised it.**

12.Row 301: “According to the… (ACMG)” – there are VCEP criteria for LDLR published in 2022 (PMID: 34906454).

**Re:** **Thank you for your comments, we have already revised it in the manuscript according to the VCEP criteria for LDLR published in 2022.**

13. Row 337: Missing space between “Thr” and “mutant”.

**Re:** **Thank you for your comments, we have already revised it.**

14.Rows 384 – 566: “Discussion”. This is NOT discussion! Presented information are rather encyclopedic information about LDLR, LDLR protein, LDLR cycle and pathogenic variants in LDLR than discussed results of this study. Why did not you discuss results of assays for Gly343Ser published here PMID: 25647241 or here PMID: 37397863 for Ala627Thr? Please, COMPLETELY revise the text.

**Re:** **Thank you for your comments, we have revised the discussion section completely.**

15.Row 520: p.Arg53fs? Why Arg53fs? This is the reason: PMID: 34786791? In my opinion, you overwrote (copy/pasted) your older text.

**Re: Very sorry, we accidentally pasted our older text (PMID: 34786791). We apologize for our carelessness and we have already revised it.**

16.Row 529: We → we

**Re:** **Thank you for your comments, we have already revised it.**

17.Row 548: Severity → severity

**Re:** **Thank you for your comments, we have already revised it.**

18.Row 714: Check the reference [39].

**Re:** **Thank you for your comments, due to a complete revision of the discussion, the content citing reference [39] has been deleted.**

19.Row 822: Table 1, Head of second column: ago → Age

**Re:** **Thank you for your comments, we have already revised it.**

20.Comment: It is necessary to mention that the authors are minimally oriented in the field of LDLR or FH. Errors on line 93 or 264 are clear evidence of this. It is necessary to completely revise the body of the manuscript. Please clearly explain how the errors on Rows 218 and 520 occurred.

**Re: Very sorry, we deeply apologize for the oversight of pasting content from our previous publications during the manuscript preparation process. We have made the necessary corrections and have thoroughly revised the introduction, discussion and conclusion sections of the paper.**

Reviewer #2:

The current study is interesting with novel and important findings. The authors used a sound methodological approach with adequate results and discussion. Here are some comments for improving the manuscript:

In the abstract:
-In materials and methods section – please mention the number of family members subjected to Sanger sequencing.

**Re:** **Thank you for your comments, we have mentioned the number of family members subjected to Sanger sequencing in materials and methods section.**

-In results section - please replace “Western blotting was showed that….” with “Western blotting has showed that……”

**Re:** **Thank you for your comments, we have already revised it.**

-In conclusion section – please replace “This study show….” with “This study shows……”

**Re:** **Thank you for your comments, we have thoroughly revised the conclusion section of the manuscript.**

In methods:
-P.12 Line 128: The line “It will start in July 2022 and end in December 2023” needs modification and to be in the past tense.

**Re:** **Thank you for your comments, but the line actually was not mentioned in the manuscript.**

-Please separate the exome sequencing section from the sanger sequencing one.

**Re:** **Thank you for your comments, we have divided the exome sequencing section and the Sanger sequencing section into two parts.**

-Please mention the number of family members subjected to Sanger sequencing and if there any other investigations have been done to them or not.

**Re:** **Thank you for your comments, fourteen family members subjected to Sanger sequencing have been mentioned in methods. And all of them performed investigations including routine physical examination, blood biochemistry, electrocardiogram, carotid ultrasound, renal artery ultrasound, echocardiography, and coronary CTA, which has been mentioned in the Research subject section.**
