# Supplementary material for: Analysis of low-density lipoprotein receptor gene mutations in a family with familial hypercholesterolemia
Source: PLoS One. 2024 Oct 11;19(10):e0310547. doi: 10.1371/journal.pone.0310547 (PMC11469539; doi:10.1371/journal.pone.0310547)
Supplement: Attachment — Submitted filename: Response to Reviewers (1).docx [file pone.0310547.s003.docx]

**Dear editors,**

Thank you for your letter and for the reviewers’ comments concerning our manuscript entitled “Analysis of low-density lipoprotein receptor gene mutations in a family with familial hypercholesterolemia”(Submission ID number PONE-D-24-12858). Those comments are all valuable and very helpful for revising and improving our manuscript, as well as the important guiding significance to our researches. We have studied comments carefully and have made correction which we hope meet with approval. Revised portion are marked in red in the manuscript. The main corrections in the manuscript and the responds to the reviewer’s comments are listed below this letter.

We would like to express our great appreciation to you and reviewers for comments on our manuscript. If you have any questions, please do not hesitate to contact me.

With best wishes,

Yours sincerely,

Fan Lin

Jie-Wei Luo

Hong-Li

2024-8-17

Journal Requirements:

1.Please review your reference list to ensure that it is complete and correct. If you have cited papers that have been retracted, please include the rationale for doing so in the manuscript text, or remove these references and replace them with relevant current references. Any changes to the reference list should be mentioned in the rebuttal letter that accompanies your revised manuscript. If you need to cite a retracted article, indicate the article’s retracted status in the References list and also include a citation and full reference for the retraction notice.

**Re: Thank you very much for your comments, we have already revised the reference list and we have not found any retracted articles among our references. If you identify any, please inform us promptly.**

2.For studies involving third-party data, we encourage authors to share any data specific to their analyses that they can legally distribute. PLOS recognizes, however, that authors may be using third-party data they do not have the rights to share. When third-party data cannot be publicly shared, authors must provide all information necessary for interested researchers to apply to gain access to the data. (https://journals.plos.org/plosone/s/data-availability#loc-acceptable-data-access-restrictions) For any third-party data that the authors cannot legally distribute, they should include the following information in their Data Availability Statement upon submission: 1) A description of the data set and the third-party source 2) If applicable, verification of permission to use the data set 3) Confirmation of whether the authors received any special privileges in accessing the data that other researchers would not have 4) All necessary contact information others would need to apply to gain access to the data We've returned your manuscript to your account.

**Re: Thank you for your comments, regarding the third-party data ,we have added availability of data and materials in the manuscript. our mutation points data is publicly available and can be accessed through the links below： SCV002098042 (https://www.ncbi.nlm.nih.gov/clinvar/variation/183106/); SCV002098051 (https://www.ncbi.nlm.nih.gov/clinvar/variation/252101/).**

Review Comments to the Author

Reviewer #1

The authors reported variants in LDLR gene in a Chinese family with FH. In silico analysis and functional assays were perform. Some minor issues need to be addressed.

1.Row 104: Who is the proband? Male, 53-year-old Chinese Han? Or Fig 1a, proband = II2, female!?

**Re: Very sorry, the proband is indeed II-2 in Fig 1a, a female. We apologize for our mistake and have already revised it.**

2.Row 179 petri dish → Petri dish.

**Re:** **Thank you for your comments, we have already revised it.**

3.Rows 312 - 313 …have both been → …have been both.

**Re:** **Thank you for your comments, we have already revised it.**

4.Row 315: …is classified under the PS3 criteria… I do not understand, what author want to say. G1027A is classified as Pathogenic (modified) for Familial Hypercholesterolemia by applying evidence codes (PM2, PP3, PS3, PP4, PS4, PP1_Strong, PM5, PM3, BS4) according to VCEP (<https://erepo.clinicalgenome.org/evrepo/ui/interpretation/2fc03ee7-785f-44c9-bac0-10c93ad52227>).

**Re:** **Thank you for your comments, we have already revised it.**

5.Row 316: …is classified as PS4 – the same as Row 315. I do not understand, what author want to say. PS4 (The prevalence of the variant in affected individuals is significantly increased compared to the prevalence in controls) is only one of VCEP criteria met.

**Re:** **Thank you for your comments, we have already revised it.**

6.Row 337: …Pur, … → …Puro,…

**Re:** **Thank you for your comments, we have already revised it.**

7.Row 354: Missing space between “Thr” and “mutant”.

**Re:** **Thank you for your comments, we have already revised it.**

8.Row 369: …of the LDLR. Maybe for clarify … of the mutant (or mutated) LDLR.

**Re:** **Thank you for your comments, we have already revised it.**

9.Row 382: Rredictprotein → Predictprotein

**Re:** **Thank you for your comments, we have already revised it.**

10.Row 389: LDLR-deficient disease? Please rephrase this part.

**Re:** **Thank you for your comments, we have already revised it.**

11.Row 553: In my opinion, Gly + Ser + Ala +Thr is not necessary to clarify.

**Re:** **Thank you for your comments, we have already deleted it.**

12.Row 615: Please check reference 11 and 16 = duplicity.

**Re:** **Thank you for your comments, we have already revised the reference list.**

Row 620: Please check reference 13 and 17 = duplicity.

**Re:** **Thank you for your comments, we have already revised the reference list.**

Row 669: Strom → Strøm.

**Re:** **Thank you for your comments, we have already deleted it.**

Reviewer #2: The authors have responded adequately for all the requested comments and the manuscript is suitable for publishing in its current form.

**Re: Very thank you for your comments.**
